# Supplementary material for: Phototaxis of Chlamydomonas arises from a tuned adaptive photoresponse shared with multicellular Volvocine green algae
Source: Phys Rev E. Author manuscript; Available in PMC 2024 Jun 12. (PMC7616094; doi:10.1103/PhysRevE.107.014404)
Supplement: Appendix [file EMS196596-supplement-Appendix.pdf]

APPENDIX A: DETAILS OF THE ITERATED MAP FROM SOLUTION OF THE INITIAL VALUE PROBLEM

Here we provide details of the derivation of the iterated map for phototurns based on explicit solution of the initial value problem for the adaptive response. For conciseness we fix the eyespot position at $\kappa = 0$ and set the time delay $\tau_d = 0$. We start from the dynamics (52), rewritten for each full turn

$n \geqslant 0$ as

$$\alpha H_T^{(n)} + H^{(n)} = \begin{cases} P^* \cos\phi \sin T; & n\pi \leqslant T < (n+1)\pi, \quad n \text{ even}, \\ 0; & n\pi \leqslant T < (n+1)\pi, \quad n \text{ odd}, \end{cases} \tag{A1}$$

and

$$\beta P_T^{(n)} + P^{(n)} = \begin{cases} P^* \cos\phi \sin T - H^{(n)}; & n\pi \leqslant T < (n+1)\pi, \quad n \text{ even}, \\ -H^{(n)}; & n\pi \leqslant T < (n+1)\pi, \quad n \text{ odd}. \end{cases} \tag{A2}$$

Solving this in a piecewise fashion we obtain

$$H^{(n)} = P^* \cos\phi \frac{\alpha}{1+\alpha^2} \times \begin{cases} C_n e^{-(T-n\pi)/\alpha} + \frac{1}{\alpha}\sin T - \cos T; & n\pi \leqslant T < (n+1)\pi, \quad n \text{ even}, \\ C_n e^{-(T-n\pi)/\alpha}; & n\pi \leqslant T < (n+1)\pi, \quad n \text{ odd}, \end{cases} \tag{A3}$$

where $C_n = (1 - r^{n+1})/(1-r)$ and $r = e^{-\pi/\alpha}$. Continuity of H at the end of each light interval can be verified by noting that $H^{(n)}(T = (n+1)\pi) \propto rC_n + 1$ for even n, while $H^{(n)}(T = n\pi) \propto C_{n+1}$ for the subsequent odd n, and observing that $1 + rC_n = C_{n+1}$.

The solution for the photoresponse variable can be expressed as $P^{(n)} = P^* \cos\phi\, \tilde{P}^{(n)}$, where

$$\tilde{P}^{(n)} = \begin{cases} \Lambda_1 D_n e^{-(T-n\pi)/\beta} - \Lambda_2 C_n e^{-(T-n\pi)/\alpha} + \Lambda_3 \sin T + \Lambda_4 \cos T; & n\pi \leqslant T < (n+1)\pi, \quad n \text{ even}, \\ \Lambda_1 D_n e^{-(T-n\pi)/\beta} - \Lambda_2 C_n e^{-(T-n\pi)/\alpha}; & n\pi \leqslant T < (n+1)\pi, \quad n \text{ odd}, \end{cases} \tag{A4}$$

with $D_n = (1 - q^{n+1})/(1-q)$, $q = e^{-\pi/\beta}$, and

$$\Lambda_1 = \frac{\alpha\beta}{(1+\beta^2)(\alpha-\beta)}, \quad \Lambda_2 = \frac{\alpha^2}{(1+\alpha^2)(\alpha-\beta)}, \quad \Lambda_3 = \frac{\alpha(\alpha+\beta)}{(1+\beta^2)(1+\alpha^2)}, \quad \Lambda_4 = \frac{\alpha(1-\alpha\beta)}{(1+\beta^2)(1+\alpha^2)}. \tag{A5}$$

Since n represents the number of half-turns, with even(odd) values for the illuminated(shaded) periods, we integrate for each value of $n \geqslant 0$ to obtain

$$\phi_{n+1} = \phi_n - P^* \cos\phi \int_{n\pi}^{(n+1)\pi} \tilde{P}^{(n)} \sin T\, dT. \tag{A6}$$

This has the form of Eq. (57), but with an n-dependent ξ_n,

$$\xi_n = -P^* \Xi_n(\omega_3), \tag{A7}$$

where

$$\Xi_n(\omega_3) = \begin{cases} \beta^2 \Lambda_1 D_n (q+1) \\ -\alpha^2 \Lambda_2 C_n (r+1) + \frac{\pi}{2}\Lambda_3, & n \text{ even}, \\ -\beta^2 \Lambda_1 D_n (q+1) + \alpha^2 \Lambda_2 C_n (r+1), & n \text{ odd}. \end{cases} \tag{A8}$$

From the general structure of the iterated map, it is clear that the larger is ξ_n the larger the angular change within a given half-turn. It is of interest then to consider the average $\bar{\Xi} = (\Xi_0 + \Xi_1)/2$ over the first two half-turns, which gives the average coefficient

$$\bar{\xi} = -P^* \bar{\Xi}(\omega_3). \tag{A9}$$

The quantity $\bar{\Xi}$ can be interpreted as the initial photoresponse function analogous to the steady-state response embodied in the amplitude $G(\omega_3)$ and phase χ_0 in Eq. (31). The functions $G(\omega_3)\cos\chi_0(\omega_3)$ and $\bar{\Xi}$ are compared in Fig. 22, where we see that the transient response function $\bar{\Xi}$ is about 10% higher at its peak, a feature that can be attributed to the fact that the hidden variable H has not yet built up to its steady value. But the two functions are otherwise remarkably similar, indicating the accuracy of the steady-state approximation.

More generally, the coefficients Ξ_n exhibit an oscillating decay with n, converging as $n \to \infty$ to

$$\Xi_\infty(\omega_3) = \begin{cases} \beta^2 \Lambda_1 \frac{1+q}{1-q} - \alpha^2 \Lambda_2 \frac{1+r}{1-r} + \frac{\pi}{2}\Lambda_3, & n \text{ even}, \\ -\beta^2 \Lambda_1 \frac{1+q}{1-q} + \alpha^2 \Lambda_2 \frac{1+r}{1-r}, & n \text{ odd}. \end{cases}$$

The connection to the steady-state approximation is obtained by considering the average $\bar{\Xi}_\infty(\omega_3)$ over the light and dark

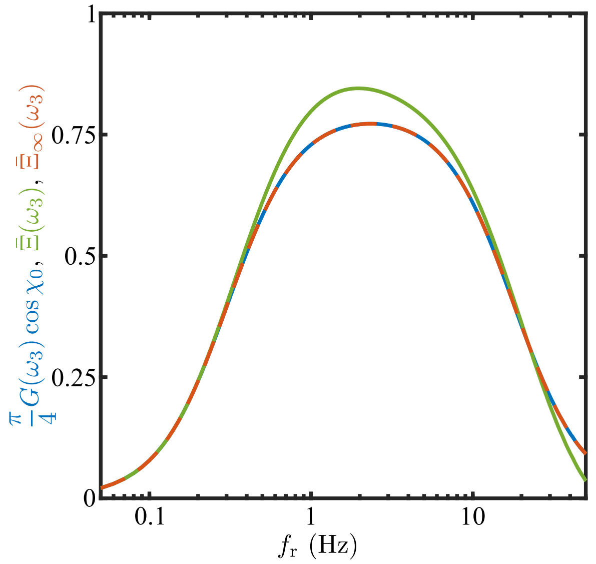

FIG. 22. Response functions. For $\kappa = \tau_d = 0$, the graph compares the steady-state response function $(\pi/4)G(\omega_3)\cos\chi_0$ in Eq. (31) (dashed blue), the initial average response $\bar{\Xi}(\omega_3)$ in Eq. (A9) (green) and the $n \to \infty$ limit of the transient response $\bar{\Xi}_\infty(\omega_3)$ (dashed red), as functions of $f_r = \omega_3/2\pi$.

cycles (hence over even and odd values of n), where one finds $\bar{\Xi}_\infty(\omega_3) = (\pi/4)\Lambda_3$, or

$$\bar{\Xi}_\infty(\omega_3) = \frac{\pi}{4}G(\omega_3)\cos(\chi_0), \tag{A10}$$

completely consistent with the steady-state analysis (58).

In the animation shown in Supplemental Video 2 [44] of the cell reorientation dynamics we evolve the iterated map using the $\{\xi_n\}$ and linearly interpolate between the values ϕ_n to obtain a smooth function of time.

APPENDIX B: DETAILS OF CONTINUOUS MODEL

Using the solution of the initial value problem we can compute the continuous approximation to the evolution equation for ϕ by integrating over the fast photoresponse variables within a turn. From the governing equation $\phi_T = -P^* \cos\phi\, \tilde{P}^{(n)}(T)\sin T$, we obtain

$$\Phi(T) = 2\tan^{-1}(e^{-P^* Q^{(n)}(T)}), \tag{B1}$$

where

$$Q^{(n)}(T) = \int_0^T \tilde{P}^{(n)}(T')\sin T' dT'. \tag{B2}$$

From Eq. (A4) we find

$$Q^{(n)}(T) = \begin{cases} -\beta\Lambda_1 D_n e^{-\frac{T-n\pi}{\beta}}(\sin T + \beta\cos T) + \alpha\Lambda_2 C_n e^{-\frac{T-n\pi}{\alpha}}(\sin T + \alpha\cos T) \\ \quad +\Lambda_3\left(\frac{T}{2} - \frac{\sin 2T}{4}\right) + \Lambda_4\frac{\sin^2 T}{2} - \Lambda_3\frac{n\pi}{4} + \beta^2\Lambda_1 - \alpha^2\Lambda_2, & n\pi \leqslant T < (n+1)\pi,\ n\,\text{even}, \\ -\beta\Lambda_1 D_n e^{-\frac{T-n\pi}{\beta}}(\sin T + \beta\cos T) + \alpha\Lambda_2 C_n e^{-\frac{T-n\pi}{\alpha}}(\sin T + \alpha\cos T) \\ \quad +\Lambda_3\frac{(n+1)\pi}{4}, & n\pi \leqslant T < (n+1)\pi,\ n\,\text{odd}. \end{cases} \tag{B3}$$

As shown in Fig. 23, the function $Q^{(n)}(T)$ typically increases monotonically with T, exhibiting small oscillations around an interpolant that grows nearly linearly with time. These magnitudes of these oscillations vary between the light and dark halves of each turn. To quantify this asymmetry we compute the values $Q^{(n)}(n\pi)$ at the start of each half-turn,

$$Q^{(n)}(n\pi) = \begin{cases} \beta^2\Lambda_1(1-D_n) - \alpha^2\Lambda_2(1-C_n) + \Lambda_3\frac{n\pi}{4}, & n\ \text{even}, \\ \beta^2\Lambda_1 D_n - \alpha^2\Lambda_2 C_n + \Lambda_3\frac{(n+1)\pi}{4}, & n\ \text{odd}, \end{cases}$$

and the gradients $\mathcal{Q}_n = [Q^{(n+1)}((n+1)\pi) - Q^{(n)}(n\pi)]/\pi$ of line segments connecting the half-turn endpoints. One can easily show that $\mathcal{Q}_n = \Xi_n/\pi$. The light-dark variation of these slopes serves as a measure of the smoothness of the reorientation dynamics, and from the first two values $\mathcal{Q}_0$ and $\mathcal{Q}_1$ we define two relevant quantities: the *strength* of the initial response as measured by the average of the slopes of the first two line segments $\bar{\mathcal{Q}} = (\mathcal{Q}_0 + \mathcal{Q}_1)/2 = \bar{\Xi}/\pi$, and its *smoothness*, as measured by the ratio $\mathcal{Y} = \mathcal{Q}_1/\mathcal{Q}_0$.

If, as in Fig. 23, we approximate $Q^{(n)}(T)$ by the line $\bar{\mathcal{Q}}T$, then the reorientation dynamics (B2) takes the simple form

$$\Phi(T) = 2\tan^{-1}(e^{-P^*\bar{\mathcal{Q}}T}), \tag{B4}$$

from which we identify the characteristic relaxation time τ_x (in physical units) analogous to Eq. (62),

$$\tau_x = \frac{1}{|\omega_1^*|\bar{\mathcal{Q}}} = \frac{\tilde{\zeta}_r}{|\mathcal{T}_{\rm p}^*|\bar{\mathcal{Q}}}. \tag{B5}$$

Finally, we explore the space of reorientation dynamics by probing $Q^{(n)}(T)$ through its dependency on parameters α and β. Our strategy is to observe how the quantities $\bar{\mathcal{Q}}(\alpha,\beta)$ [Fig. 24(a)] and $\mathcal{Y}(\alpha,\beta)$ [Fig. 24(b)], which are also functions of α and β, and essentially describe the curve's shape, vary. First, we make the observation that the (α,β) pairs acquired from micropipette experiments [step-up and frequency response; Fig. 12(b)] lie in the high-slope area ($\bar{\mathcal{Q}} = 0.27$) of the $\bar{\mathcal{Q}}(\alpha,\beta)$ function [Figs. 24(a) and 24(c)]. We also observe that the same data lie in an area of relatively moderate symmetry ($\mathcal{Y} \approx 0.34$) of the $\mathcal{Y}(\alpha,\beta)$ function [red markers

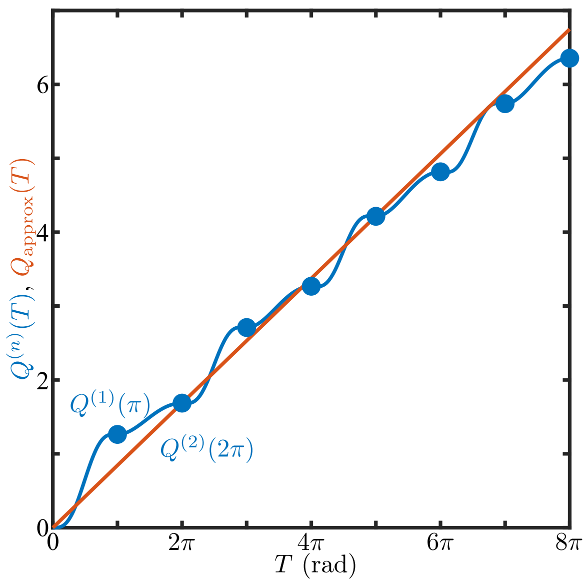

FIG. 23. The functions $Q(T)$. The function $Q^{(n)}(T)$ (blue), defined in a piecewise manner between endpoints (blue circles), can be approximated as a straight line $Q_{\rm approx}(T) = \bar{\mathcal{Q}}T$ (red). Both $Q^{(n)}(T)$ and $\bar{\mathcal{Q}}$ were computed with the experimentally derived $\tau_r = 0.009$ s and $\tau_a = 0.524$ s. The value of f_r was taken to be 1.67 Hz. The value of $\bar{\mathcal{Q}}(\omega_3, \tau_r, \tau_a)$ was calculated to be 0.268.

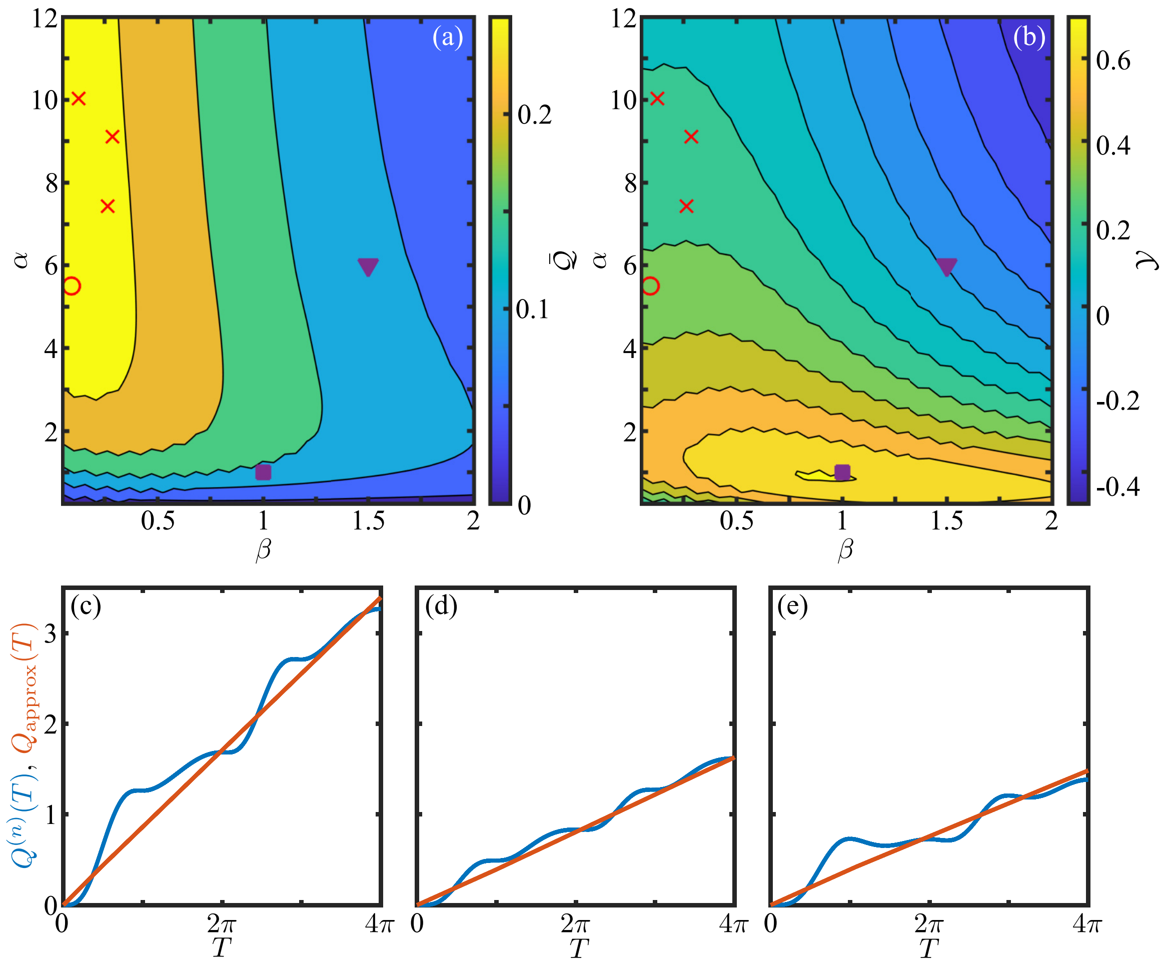

FIG. 24. Contour plot maps of $\bar{\mathcal{Q}}$ (a) and $\mathcal{Y}$ (b) with (α, β) pairs acquired from micropipette experiments shown with red markers (step up as "x" and frequency as "o"). (c)–(e) Plots of $Q^{(n)}(T)$ (blue line) and linear approximation $Q_{\rm approx}(T) = \bar{\mathcal{Q}}T$ (red line) for three (α, β) value pairs shown in panels (a) and (b) as circle, square, and triangle, respectively. (c) is based on experimental data (open red circle), (d) corresponds to $\mathcal{Y} \approx 0.7$ (solid purple square), and (e) corresponds to $\mathcal{Y} \approx 0$ (solid purple triangle).

in Fig. 24(b)], as opposed to the extreme cases of highest symmetry, i.e., $\mathcal{Y} \approx 0.7$ [solid purple square in Figs. 24(b) and 24(d)] and lowest symmetry, i.e., $\mathcal{Y} \approx 0$ [solid purple triangle in Figs. 24(b) and 24(e)].
